# Weight Loss Effect of GLP-1 RAs With Endoscopic Bariatric Therapy and Bariatric Surgeries

**DOI:** 10.1210/jendso/bvad129

**Published:** 2023-10-19

**Authors:** Ahmad Imam, Hussam Alim, Mohammad Binhussein, Abdulrahman Kabli, Husam Alhasnani, Abdullah Allehyani, Ammar Aljohani, Ahmad Mohorjy, Abdullah Tawakul, Mohammed Samannodi, Wael Taha

**Affiliations:** Department of Internal Medicine, Umm Al-Qura University, Makkah 24381, Saudi Arabia; Department of Internal Medicine, Umm Al-Qura University, Makkah 24381, Saudi Arabia; Department of Internal Medicine, Umm Al-Qura University, Makkah 24381, Saudi Arabia; Department of Internal Medicine, Umm Al-Qura University, Makkah 24381, Saudi Arabia; Department of Internal Medicine, Umm Al-Qura University, Makkah 24381, Saudi Arabia; Department of Internal Medicine, Umm Al-Qura University, Makkah 24381, Saudi Arabia; Department of Internal Medicine, Umm Al-Qura University, Makkah 24381, Saudi Arabia; Department of Internal Medicine, Umm Al-Qura University, Makkah 24381, Saudi Arabia; Department of Internal Medicine, Umm Al-Qura University, Makkah 24381, Saudi Arabia; Department of Internal Medicine, Umm Al-Qura University, Makkah 24381, Saudi Arabia; Department of Internal Medicine, Division of Endocrinology, Wayne State University, Detroit, MI 48201, USA

**Keywords:** obesity, weight loss, GLP-1 receptor agonist, bariatric surgeries, endoscopic bariatric therapy, liraglutide

## Abstract

**Background:**

Different treatment modalities are available for obesity management, including lifestyle changes, pharmacotherapy, endoscopic interventions, and surgeries. Limited evidence is available on the weight loss effect of combining glucagon-like peptide 1 receptor agonists (GLP-1 RAs) with endoscopic bariatric therapy (EBT) and bariatric surgeries (BS).

**Objectives:**

In this systematic review, we compared the weight loss effect and metabolic changes of combining GLP-1 RAs with EBT and BS.

**Methods:**

Literature searches were performed in the Cochrane Database of Systematic Review, Cochrane Central Register of Controlled Trials, Embase, PubMed, Google Scholar, and PRISMA databases. Only randomized control trials and retrospective studies were included.

**Results:**

A total of 11 studies was included. Nine studies compared BS with and without liraglutide and 2 compared EBT with and without liraglutide. Adding liraglutide to EBT or BS provided significant weight loss when compared with EBT or BS alone. When changes in weight were compared across the studies, EBT with liraglutide showed a weight loss effect comparable to the net weight loss (ie, nadir weight loss after BS-regained weight) achieved following BS alone.

**Conclusion:**

This review showcases a promising approach for managing obesity that combines GLP-1 RAs with EBT. This approach is expected to achieve shorter hospital stays, fewer side effects, and longer term weight loss benefits than BS alone. However, additional prospective studies with higher quality, more consistent outcome measures for weight loss and metabolic changes are needed to further evaluate the approach.

## Background

Obesity, an increasingly prevalent health problem worldwide, affects more than one-third of the global population [1]. The condition has been associated with increased morbidity and mortality as well as lower life expectancy [2]. However, losing 5% to 15% of one's body weight has been shown to improve cardiometabolic risk and obesity-related complications [3]. Treatment modalities currently used to manage obesity include lifestyle changes, pharmacotherapy, surgery, and endoscopic interventions.

Lifestyle changes, as the first-line treatment modality for obesity, include exercise, changes in diet, and behavioral therapy [4]. Although lifestyle changes can reduce patients’ weight by 8% to 10% in approximately 30 weeks [4], maintaining a healthy lifestyle in the long term is usually difficult, and the approach is associated with not only high rates of failure but also weight regain.

When lifestyle changes fail, pharmacological treatment can be used to manage obesity. The pharmacological treatment of obesity is recommended for patients with a body mass index (BMI) > 30 kg/m^2^ or >27 kg/m^2^ with comorbidities. There are different pharmacological options for obesity management, including orlistat, naltrexone-bupropion, phentermine-topiramate, and glucagon-like peptide 1 receptor agonists (GLP-1 RAs) [5, 6]. Another pharmacological treatment, lorcaserin, was withdrawn from the market by the US Food and Drug Administration (FDA) in 2020 because of the increased risk of cancer among its users [7].

GLP-1 RAs are the most commonly used medications for managing obesity. They stimulate insulin secretion, slow gastric emptying while increasing satiety, and reduce postprandial glucagon and food intake [8]. At present, only 2 GLP-1 RAs have been approved by the FDA for managing obesity: liraglutide, approved in 2014, and semaglutide, approved in 2020 [9]. Studies have shown that GLP-1 RAs can reduce patients’ weight by up to 15% over the course of 12 months [6, 10]. Even so, the weight loss response to GLP-1 RAs varies from patient to patient, some of whom experience a plateau effect following the long-term use of the medication [11-13] The most commonly reported side effects of GLP-1 RAs are gastrointestinal symptoms [6, 10].

Bariatric surgery (BS), another treatment modality for managing obesity, is indicated for patients with a BMI > 40 kg/m^2^ and for patients with a BMI of 35 to 40 kg/m^2^ with comorbidities [14]. The most common types of BS are vertical sleeve gastrectomy (VSG), gastric band (GB) surgery, and Roux-en-Y gastric bypass (RYGB) surgery. Of these, RYGB surgery is the most effective, despite also being associated with a higher rate of complications. Indeed, various complications and drawbacks are associated with BS, including leaks at the surgical site, bleeding, stenoses, venous thromboembolism, and insufficient weight loss or even subsequent weight regain.

The final treatment modality for managing obesity is endoscopic bariatric therapy (EBT), including intragastric balloons (IGBs), aspiration therapy, and endoscopic sleeve gastroplasty (ESG). All of these forms of EBT can be performed in a 1-day setting and result in fewer complications than other invasive surgical options [15]. Although such treatments continue to evolve, they are currently linked to significantly high rates of failure [16-19].

Considering all of these treatments, there is an immense need for a treatment modality for managing obesity that is relatively noninvasive, is effective in both the short and long term, has high rates of success, and involves few side effects. One such modality may be combining GLP-1 RAs with EBT or BS [20-27]; however, evidence of that modality's effect on weight loss is currently limited.

## Primary Objective

To compare the weight loss effect of GLP-1 RAs when combined with EBT or BS.

## Secondary Objectives

To compare the metabolic changes of GLP-1 RAs when combined with EBT or BS.

## Methods

### Search Strategy and Protocol

To identify articles for our systematic review, we conducted literature searches in the Cochrane Database of Systematic Reviews [28], Cochrane Central Register of Controlled Trials [29], Embase [30], PubMed, Google Scholar, and PRISMA databases. The following terms were used to search for relevant articles: *“*laparoscopic sleeve gastrectomy OR bariatric surgery OR metabolic surgery OR endoscopic bariatric therapy OR intragastric balloon OR post-bariatric surgery OR prior bariatric surgery endoscopic sleeve gastroplasty OR ESG AND liraglutide OR GLP-1 analogues OR GLP-1 agonists AND type 2 diabetes OR obesity OR weight reduction OR weight loss OR excessive weight regain.”

To reduce publication bias in our systematic review, we reviewed the reference lists of the articles that resulted from our searches as well as other major reviews and added a second reviewer. Afterward, the abstracts and citations of all relevant articles were screened.

### Article Selection Criteria

To be included in our systematic review, articles had to be (1) written in English and (2) present prospective or retrospective study design that were (3) conducted with human participants (4) who were at least aged 18 years and (5) involved comparing the weight loss change in patients who received any form of GLP-1 RAs following BS or EBT. Conversely, articles were excluded if they were (1) not written in English, (2) presented studies involving patients aged younger than 18 years, (3) presented studies using GLP-1 RAs without any surgical or endoscopic intervention, or (4) did not report the weight loss effect of GLP-1 RAs separately.

### Quality Assessment

The checklists recommended by the National Institutes of Health were used to assess the quality of the articles that were eligible for our systematic review [31].

### Data Extraction

Two independent reviewers reviewed all of the selected articles and extracted relevant data directly into an evidence table. Extracted data included information about the authors, publisher, study quality, study design, study method (ie, allocation, duration, blinding, numbers enrolled, follow-up rate, biases, and confounders), participants’ characteristics (ie, sex, age, demographic variations, and geographical regions), interventions used (eg, liraglutide), and a summary of the study

## Results

### Flow Chart of the Article Selection Process

The initial literature search returned 16 548 studies that matched the initial search criteria; of these, 16 538 articles were excluded after the exclusion criteria were applied. Thus, 11 articles were included in the review: 3 randomized controlled trials (RCTs) and 8 retrospective studies. Nine of the studies compared BS with and without liraglutide, whereas 2 compared EBT with and without liraglutide. The studies were conducted in Brazil, Canada, India, Spain, Saudi Arabia, Switzerland, the United Arab Emirates, and the United Kingdom between 2013 and 2023. The total number of participants in all included studies was 914. The types of BS performed on the participants were RYGB surgery, gastric sleeve surgery, and laparoscopic sleeve gastrectomy (LSG). Meanwhile, the endoscopic procedures included IGB insertion and ESG. The baseline characteristics of each article, the surgical procedures, and the medications used are listed in Table 1.

**Table 1.** Articles’ baseline characteristics, surgical procedures, and medications used

Article	Author	Publication year (country)	Study type	Sample size (age)	Female	Surgery	Medications (dose)	Time since surgery	Outcomes	Follow-up
**1.**	Mok et al [32]	2023 (UK)	RCT	70 (47.6 ± 10.7)	74%	GS and RYGB	Liraglutide (3 mg)	12 mo	%TWL, metabolic changes	24 wk
**2.**	Jensen et al [33]	2023 (Switzerland)	Retrospective	50 (43)	82%	RYGB, GS, GB	Liraglutide (1.8 and 3 mg), subcutaneous semaglutide (1 mg), oral semaglutide (14 mg)	72 mo	%TWL	6 mo
**3.**	Thakur et al [24]	2020 (India)	RCT	23 (42.9 ± 10.9)	52.1%	LSG	Liraglutide (3 mg)	6 wk	%TWL, %EWL, and metabolic changes	6 mo
**4.**	Miras et al [26]	2019 (UK)	RCT	80 (56)	58.7%	RYGB and VSG	Liraglutide (1.8 mg)	3.8-4 y	%TWL and metabolic changes	26 wk
**5.**	Wharton et al [23]	2019 (Canada)	Retrospective	117 (51.2 ± 9.4)	87.2%	GB, GS, and RYGB	Liraglutide (3 mg)	4-10 y	%TWL	7.6 ± 7.1 mo
**6.**	Suliman et al [25]	2019 (UAE)	Retrospective	188 (38)	75.0%	VSG and RYGB	Liraglutide (3 mg)	4 y	%TWL	16-42 wk
**7.**	Rye et al [27]	2018 (Canada)	Retrospective	33 (49.6 ± 8.3)	95.0%	GB, GS, and RYGB	Liraglutide (3 mg)	76 mo	%WL	16-28 wk
**8.**	Gorgojo-Martínez et al [34]	2016 (Spain)	Retrospective	164 (53.0 ± 9.0)	66.7%	GB, GS, RYGB, and BPD	Liraglutide (1.6 mg)	5.2 y	WL	12–24 mo
**9.**	Pajecki et al [35]	2013 (Brazil)	Retrospective	15 (47.2 ± 12.5)	66.7%	LSG, GB, RYGB, and BPD	Liraglutide 1.2 to 3.0 mg	5.3 ± 3.3 y	%EWL	8.6 ± 7.3 mo
**10.**	Badurdeen et al [20]	2020 (Brazil)	Retrospective	66 (NM)	NM	ESG	Liraglutide (3 mg)	5 mo	%TWL	12 mo
**11.**	Mosli et al [22]	2017 (KSA)	Retrospective	108 (33.7)	71.2%	Intragastric balloon (IGB)	Liraglutide (3 mg)	1 mo	MWL	6 mo

Abbreviations: %EWL, percentage of excess body weight loss; %TWL, percentage of total weight loss; BPD, biliopancreatic diversion; ESG, endoscopic sleeve gastroplasty; GB, gastric band; GS, gastric sleeve; IGB, intragastric balloon; KSA, Kingdom of Saudi Arabia; LSG, laparoscopic sleeve gastrectomy; MWL, mean weight loss, adverse effects; NM, not mentioned; RCT, randomized control trial; RYGB, Roux-en-Y gastric bypass; S/Q, subcutaneous; UAE, United Arab Emirates; VSG, vertical sleeve gastrectomy.

### Weight Loss Effect and Metabolic Changes: BS and Liraglutide

In 1 RCT (ie, BARI-OPTIMISE) conducted earlier in 2023, Mok et al [32] examined the efficacy of using 3.0 mg liraglutide along with a 500-kcal deficit among patients who did not achieve adequate body weight loss (ie, >20%) after at least 12 months following LSG or RYGB surgery. In their study, 70 such patients were randomized to receive either liraglutide plus a 500-kcal deficit or placebo plus a 500-kcal deficit and were followed up for 6 months.

The liraglutide group showed a greater reduction in mean percentage body weight than the placebo group (−8.82 vs −0.54, respectively; *P* < .001). The mean difference in the percentage body weight change between the groups was 8.03 (*P* < .01); 71.9% of the liraglutide group lost 5% or more of their body weight compared with only 8.8% of the placebo group. The liraglutide group presented favorable metabolic changes compared with the placebo group, including lower fasting blood sugar, glycated hemoglobin (HbA1c) levels, low-density lipoprotein, and both systolic and diastolic blood pressures. However, the liraglutide group also showed a higher rate of adverse events; the most commonly reported were nausea, constipation, and fatigue.

Also in 2023, Jensen et al [33] conducted a retrospective observational study to evaluate the efficacy of using either liraglutide or semaglutide to treat weight regain following BS. Fifty patients (82% female) who had experienced weight regain after the weight loss nadir at least 12 months following BS received 1 of 4 interventions: liraglutide 3 mg daily (n = 28), liraglutide 1.8 mg daily (n = 1), subcutaneous semaglutide 1 mg weekly (n = 20), or oral semaglutide 14 mg daily (n = 1). The patients were followed up for 6 months, after which 82% of them underwent proximal RYGB surgery, 10% underwent sleeve gastrectomy, 8% underwent distal RYGB surgery, and 14% underwent BS twice.

Seventy-two months after BS, patients had regained a median of 15.1% of their total body weight. After 6 months of treatment with a GLP-1 RA, they had lost 8.8% of their total body weight (*P* < .0001), corresponding to a median of 67.4% of the weight regained after BS. Total medication-specific weight loss was 7.3% and 9.8% following liraglutide treatment and semaglutide treatment, respectively. Over 6 months of treatment, 85.7% of the patients on semaglutide lost at least 5% of their total body weight compared with only 69.0% of the patients on liraglutide. Nausea and constipation were the most commonly reported side effects.

In another RCT, Thakur et al [24] recruited 23 patients, 12 of whom were female, whose mean age was 42.9 ± 10.9 years, mean BMI was 42.5 ± 5.6 kg/m^2^, and mean weight was 109.7 ± 18.5 kg. Six weeks after undergoing LSG, all patients were randomized into 2 groups; 1 group received liraglutide in increasing doses of 0.6 mg/d up to 3 mg (L-L group), whereas the other received a placebo treatment (L-P group). At follow-up after 6 months, the mean percentage of total weight loss was 28.2 ± 5.7% and 23.2 ± 6.2% (*P* = .116) in the L-L group and L-P group, respectively. The mean percentage of excess weight loss was significantly higher in the L-L group than in the L-P group (58.7 ± 14.3% vs 44.5 ± 8.6%; *P* = .043), with an intergroup difference of 14.2 ± 5.4%. Mean BMI decreased by 11.7 ± 3.5% in the L-L group and by 9.5 ± 4.0% in the L-P group (*P* = .287). Overall, the L-L group lost an additional 14% of weight on average compared with the placebo group and showed significant percent reductions in fasting blood glucose, postprandial blood glucose, and HbA1c levels—11.7%, 15.0%, and 13.2%, respectively—from the baseline. All patients in the L-L group experienced the complete resolution of their hypertension compared with only 80% in the placebo group (*P* = .08). Dyslipidemia was resolved in 71.4% of the patients in the liraglutide group compared with only 50.0% of patients in the surgery-only group; however, no significant differences in other obesity-related complications emerged between the groups. The most commonly reported side effects were nausea, vomiting, and headache, all with similar incidence between the groups.

In the remaining RCT we reviewed, Miras et al [26] randomly assigned 80 patients in 1 of 2 groups following BS. The first group (n = 53) received a daily 1.8 mg dose of subcutaneous liraglutide for 26 weeks, whereas the second group (n = 27) received a placebo; both groups also adopted a calorie-deficit diet and engaged in physical exercise. The time since BS but before starting liraglutide ranged from 3.8 to 4 years. No significant differences in baseline characteristics were evident between the groups; however, patients in the liraglutide group had a slightly higher mean HbA1c level (7.9% points, SD = 1.39) than the placebo group (7.4% points, SD = 0.75). Whereas 61 patients underwent RYGB surgery—42 in the liraglutide group and 19 in the placebo group—19 patients underwent VSG (ie, 11 in the liraglutide group and 8 in the placebo group). A total of 71 (89%) patients completed the trial for the full 26 weeks: 48 in the liraglutide group and 23 in the placebo group. At week 6, major improvements were observed in baseline body weight among patients in the liraglutide group (−2.38 kg; 95% CI, −3.26 to −1.49; *P <* .0001), which continued into week 10 (−3.71 kg; 95% CI, −4.59 to −2.82; *P <* .0001), week 18 (−4.46 kg; 95% CI, −5.34 to −3.57; *P <* .0001), and week 26 (−5.26 kg; 95% CI, −6.15 to −4.38; *P <* .0001). The effect did not appear to plateau at any point. By contrast, the change in baseline body weight in the placebo group was nonsignificant. Of the 48 patients in the liraglutide group, 22 (46%) reduced their baseline body weight by 5%, whereas only 2 of the 23 patients (9%) in the placebo group achieved the same result. Moreover, 7 patients (15%) in the liraglutide group lost at least 10% of their baseline body weight, whereas 2 (4%) lost 15% or more. Changes in patients’ baseline weight up to week 26 did not appear to be significantly impacted by the type of BS. By week 26, the mean HbA1c level in the liraglutide group had dropped significantly (1.22% points, *P* = .0001) compared with the level in the placebo group (0.43% points, *P* = .17). Nausea, diarrhea, and constipation were the most commonly reported side effects from liraglutide.

In Wharton et al's [23] retrospective study, 117 patients, all of whom had undergone BS, received liraglutide. They were divided into 3 groups according to the type of BS: RYGB surgery (45.3%), gastric balloon surgery (42.7%), and gastric sleeve surgery (5%). Patients’ mean age was 51.2 ± 9.4 years, their mean BMI before BS was 49.7 ± 12.1 kg/m^2^, and most patients were female (87.2%). Following BS, patients’ mean weight loss was 40.7 ± 25.0 kg, for a 28.0% drop in weight on average. Before starting liraglutide, patients had regained 21.2 ± 16.9 kg (58.6%) of their maximum weight loss and had a mean BMI of 42.5 ± 9.6 kg/m^2^. The liraglutide dose was titrated to reach 3 mg once daily, a dose that 62% of the patients could tolerate. Liraglutide was taken for an average of 8 years (7.8 ± 5.7 years; interquartile range [IQR], 4-10 years) following BS. After taking liraglutide for 7.6 ± 7.1 months, patients lost an average of 5.5% ± 6.2% (6.3 ± 7.7 kg, *P <* .05) of their additional weight. The BMI of patients who had undergone RYGB surgery (39.0 ± 7.0 kg/m^2^) was lower than that of patients who had undergone GB surgery (45.4 ± 11.0 kg/m^2^) or gastric sleeve surgery (45.4 ± 9.6 kg/m^2^) before the 3 mg of liraglutide was initiated (*P <* .05). Regardless of the type of BS, the amount of weight loss was similar in all patients after receiving liraglutide (*P* > .05). Once liraglutide treatment was commenced, significant weight loss was experienced by patients as early as a month later and continued to be significant for up to 12 months regardless of the type of BS received (*P* > .05). Although no data on metabolic changes are reported in the article, the most prevalent side effect among patients was nausea.

In Suliman et al's [25] retrospective data collection study, 188 patients were enrolled who had undergone BS—sleeve gastrectomy (63%), RYGB surgery (25%), and other procedures (12%)—and received 3 mg liraglutide daily. The median time between BS and the commencement of liraglutide was 4 years, with the treatment duration ranging from 16 to 42 weeks; only 76 of the patients had received liraglutide treatment for 16 weeks and more. Overall, patients lost a median of 6 kg (2.4-9.4 kg) of body weight, or an equivalent of 6.4% (2.5%-9.7%) of their baseline weight. Weight loss was greater among patients who had undergone RYGB surgery than those who had undergone sleeve gastrectomy (5.6% vs 3.3%, *P* = .025). The authors did not provide data regarding metabolic changes, and patients most often reported nausea, vomiting, and diarrhea as side effects of liraglutide.

Rye et al [27] enrolled 33 patients who had received liraglutide following BS in a retrospective data review study. Only 20 patients met the inclusion criteria; their mean age was 49.6 ± 8.3 years, and 95% of them were female. Patients had RYGB (35%), LSG (35%), vertical banded gastroplasty (15%), and adjustable GB surgery (15%). Patients were prescribed liraglutide, titrated to reach 3.0 mg daily, if they had more than 10% weight regain from their lowest postsurgical weight, had less than 20% weight loss from their presurgical weight, or had a plateau of weight loss. The average duration between surgery and the commencement of liraglutide was 76.3 months. The median percentage of weight loss and median change in BMI was measured at weeks 16 and 28; the median weight loss was 7.1% (IQR, 5.1%-12.2%) and 9.7% (IQR, 7.8%-13.9%), respectively, whereas the median change in BMI was 3.5 kg/m^2^ (IQR, 2.2-4.6 kg/m^2^) and 4.7 kg/m^2^ (IQR, 3.7-5.6 kg/m^2^), also respectively. In a subgroup analysis, the authors compared the final weight loss at week 28 among patients who had achieved more than 5% weight loss at week 16 (ie, early responders) with participants who had not. By week 28, early responders had lost more weight and had lower BMIs than non-early responders (12.2% vs 6.4% and 4.9 kg/m^2^ vs 2.0 kg/m^2^ for weight loss and BMI, respectively). In another subgroup analysis, patients with type 2 diabetes (n = 5) were found to have lost 5.7% of their weight by week 16, with a median BMI reduction of 3.5 kg/m^2^.

By week 28, patients with type 2 diabetes had lost a median of 8.4% of their body weight and had a median BMI of 4.1 kg/m^2^. When the patients were stratified according to the indication for liraglutide, all 3 groups showed comparable drops in median weight loss and BMI in week 28. Although no other metabolic outcomes were reported, nausea was the most commonly reported side effect.

In Gorgojo-Martínez et al's study, 164 patients with diabetes and obesity who received 1.6 mg liraglutide daily for at least 2 years were enrolled. Of these, only 15 had a history of BS. The authors reviewed data retrospectively to evaluate differences in HbA1c levels and weight at 104 weeks between the BS group and non-BS group. On average, liraglutide was commenced 5.2 years after surgery, with a duration of use ranging from 12 to 24 months. Both groups exhibited significant reductions in HbA1c levels and weight (*P* < .05); HbA1c levels decreased by 0.39% and 0.67% points in the BS group and non-BS group, respectively, whereas their weight decreased by 3.4 and 3.8 kg, also respectively. However, no significant differences surfaced between the groups. The authors also found no changes in lipid profile or systolic blood pressure resulting from liraglutide use between the groups. Nausea and vomiting were the most commonly reported side effects of liraglutide.

Pajecki et al conducted a retrospective analysis of 15 patients who had failed BS and received liraglutide. All patients had less than 50% excess weight loss or less than 15% weight regain from their lowest postsurgical weight 2 years after surgery. Liraglutide was commenced 5.6 years after surgery on average, in a dose ranging from 1.2 to 3.0 mg/d and for a duration of 12.5 ± 4.7 weeks. Mean weight following the use of liraglutide decreased significantly (100.9 ± 18.3 kg vs 93.5 ± 17.4 kg, *P <* .0001), and nausea was the only reported side effect.

### Weight Loss Effect and Metabolic Changes: EBT and Liraglutide

In a retrospective study, Badurdeen et al [20] evaluated the advantages of adding liraglutide to ESG among 66 patients who had undergone ESG surgery. Of these, 30 (45.45%) started taking liraglutide 5 months after ESG. The baseline characteristics were similar in both groups (ie, ESG only and ESG plus liraglutide). The baseline BMI was 35.73 ± 1.96 kg/m^2^ in the ESG-only group and 35.87 ± 2.21 kg/m^2^ for the ESG plus liraglutide group. Compared with the ESG-only group, the ESG plus liraglutide group had a significantly high percentage of total body weight loss at 3 months (10.48 ± 1.74% vs 9.52 ± 1.88%, *P* = .037), 5 months (14.47 ± 1.49% vs 12.86 ± 2.34%, *P <* .002), 9 months (22.34 ± 1.91% vs 18.37 ± 2.15%, *P <* .001), and 12 months (25.07 ± 2.19% vs 20.17 ± 1.96%, *P <* .001). Moreover, patients in the ESG plus liraglutide group had a significantly higher reduction in their percentage of body fat at 12 months than patients in the ESG-only group (10.61 ± 1.86% vs 7.83 ± 1.23%, *P <* .001).

In another retrospective study, Mosli et al [22] compared the weight loss efficacy of adding liraglutide to an IGB in 108 patients, 64 of whom had an IGB inserted without liraglutide (ie, IGB-only), whereas 44 had an IGB inserted followed by liraglutide treatment (ie, IGB plus liraglutide). The patients’ mean age was 34.9 ± 9.8 years in the IGB-only group and 32.5 ± 8.4 years in the IGB with liraglutide group. A total of 47% of participants in the IGB-only group were female, whereas 30% in the IGB with liraglutide group were. The mean respective baseline BMI and body weight values were 37 ± 5.9 kg/m^2^ and 99.3 ± 19.9 kg in the IGB-only group and 38.5 ± 6.1 kg/m^2^ and 103.8 ± 19.1 kg in the IGB with liraglutide group. Liraglutide was commenced 1 month after the insertion of IGBs and discontinued 1 month after the IGBs were removed, for a maximum duration of 6 months. Patients were followed up at 3 and 6 months after IGB insertion. The mean weight loss at the time of IGB removal was higher in the IGB with liraglutide group than in the IGB-only group (18.5 ± 7.6 kg vs 10.2 ± 6.7 kg, *P <* .0001). Moreover, 6 months after IGB removal, mean weight loss between the IGB with liraglutide group and the IGB-only group differed significantly (4.7 ± 6 kg vs 2.7 ± 4.10 kg, *P* = .019). However, when the authors adjusted the results with clinically relevant baseline and follow-up covariates using multiple linear and logistic regression analysis, they found contradicting data. The adjusted data showed a higher mean body weight loss in the IGB-only group than in the IGB plus liraglutide group at the time of IGB removal (coefficient = 7.71; 95% CI, 4.78-10.63) and a higher probability of treatment success in the IGB-only group after 6 months (5.74; 95% CI, 1.79-188.42) than in the IGB plus liraglutide group. Furthermore, baseline BMI was found to be a significant predictor of mean body weight loss at the time of IGB removal. The authors thus concluded that adding liraglutide to the IGB insertion did not decrease the risk of weight regain 6 months after IGB removal.

### Overall Changes in Weight Following BS and EBT With and Without GLP-1 RAs

**Figure 1.**
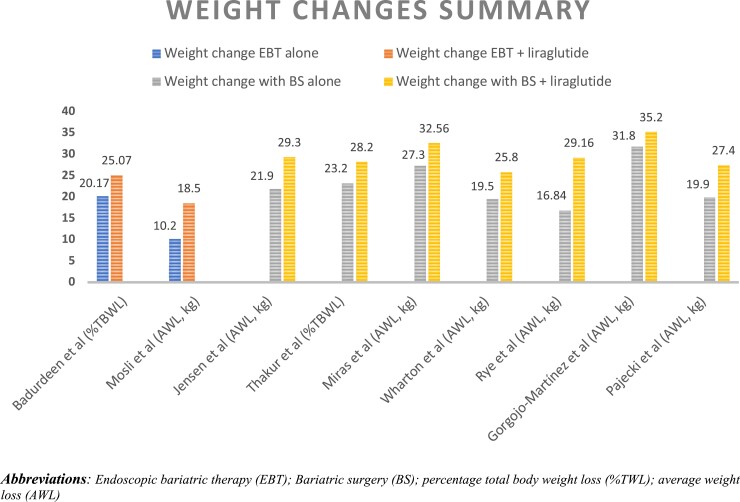
Summary of weight changes across included studies. Abbreviations: %TWL, percentage total body weight loss; AWL, average weight loss; BS, bariatric surgery; EBT, endoscopic bariatric therapy.

Weight change across the studies included in our review is plotted in Fig. 1. Mok et al's and Suliman et al's studies were excluded from this analysis because information about participants’ weight before surgery was not provided. In Fig. 1, weight regain was subtracted from the maximum weight loss after BS to evaluate the actual weight change following BS. As shown in Fig. 1, weight change was the highest in the BS with liraglutide group and lowest in EBT-only group. Interestingly, EBT with liraglutide achieved a weight loss effect comparable to BS alone. We did not conduct a meta-analysis along with our systematic review because of the limited number of studies included and their significant heterogeneity in terms of study design, type of intervention, time of intervention, duration of intervention, and follow-up time.

## Discussion

The results of the 11 articles included in our review indicate a significant weight loss effect of combining liraglutide with EBT or BS. Furthermore, when changes in weight were compared across the studies, EBT plus liraglutide showed a weight loss effect comparable to the net weight loss following BS alone (ie, nadir weight loss after BS-regained weight), as illustrated in Fig. 1. Thus, the results of the review indicate a promising new strategy for managing obesity with the combination of EBT and GLP-1 RAs. None of the included studies, however, examined metabolic changes following EBT plus liraglutide. Studies included in our review used different doses of GLP-RAs, included participants with different backgrounds in terms of disease, had inconsistent outcome measures for weight loss, and followed different methodologies with varying study durations, all of which may limit the accuracy and generalizability of our results. Furthermore, we did not perform a quantitative assessment in our review because of the small number of studies and significant heterogeneity between them.

Our results are consistent with the findings of another review that investigated the weight loss effect of GLP-1 RAs following BS [15]. That review's authors included 6 articles in their review—1 RCT and 5 retrospective studies—and found beneficial weight loss and glycemic effects with the administration of liraglutide following BS.

Amid the continued increase in the rate of obesity and associated metabolic disorders, the need for an intervention that provides sustained weight loss is mounting. Although BS can achieve significant weight loss, postsurgical complications and the risk of weight regain limit the approach's popularity in certain populations. BS is considered to have failed if the patient does not achieve or maintain less than 50% of excess weight loss after 18 to 24 months or has a BMI exceeding 35 kg/m^2^ [36]. Nonetheless, growing evidence has demonstrated the beneficial effect of BS on glycemic control independent of the amount of weight loss after BS [37-39]. The rate of failure following BS varies according to the type of procedure and significantly increases with time following surgery [40-43]; it reaches up to 18% with RYGB surgery and 44% with GB surgery [36, 40], and 50% of patients will experience postoperative weight regain after 5 years [36, 40-43]. Moreover, revisions of BS pose an even higher risk of complications [44].

EBT, having evolved considerably since the first use of the IGB in the 1980s [45], now includes different types of IGB-based treatments, gastric aspiration therapy, ESG, and gastric bypass revision [46]. Although EBT can provide greater weight loss effects than obesity pharmacotherapies, it remains less effective than BS [10, 20], even if the rate of complications associated with EBS is far lower than with BS [46, 47]. Some EBTs, including IGB-based treatments, can also be used as a bridge therapy for weight loss before BS [48]. Despite the initial weight loss effect of EBT, it is linked with nonsustained weight loss and a high rate of failure [48].

Treating obesity with medication has emerged as an alternative or additive treatment in relation to bariatric interventions. GLP-1 RAs are safe and affordable and can achieve significant weight loss. The production of GLP-1 increases primarily in response to food; it also increases after BS, which helps to maintain the weight loss effect [34]. The concentration of GLP-1 under fasting conditions has additionally been found to be similar before and after BS [26]. By extension, the addition of GLP-1 RAs has been shown to increase the fasting concentrations of GLP-1 and facilitate appetite suppression and weight loss and to improve insulin sensitivity [26]. Moreover, GLP-1 RAs have shown extended weight loss effects both with and without surgery [23, 24]. Thus, combining GLP-1 RAs with BS can be expected to improve long-term weight loss and mitigate the outcomes of metabolic disorders. Liraglutide and semaglutide are the only GLP-1 RAs currently approved by the FDA for managing obesity. Although other weekly GLP-1 RAs, including tirzepatide, have shown significant weight loss effects, they have not been approved by the FDA for managing obesity [35]. Moreover, even if tirzepatide is expected to be approved for weight loss, it may be too expensive for some patients compared with other GLP-1 RAs. However, with the improved weight loss effects achieved by new GLP-1 RAs, the combination of GLP-1 RAs with EBT can be expected to provide better weight loss and metabolic outcomes along with less adverse risks.

### Limitations of Studies Examining BS Plus Liraglutide

Mok et al [32] reported significant favorable outcomes in the mean percentage of weight loss and metabolic changes for liraglutide among patients who were also prescribed a 500-kcal deficit. However, the authors recruited only participants with suboptimal postprandial GLP-1 response, and most were Caucasian females, which limits the generalizability of their findings. Thus, the approach's effect on participants of other ethnicities and with normal postprandial GLP-1 responses need to be studied. Moreover, because the study's participants were followed up for 24 weeks only with no reported weight loss plateau, the longer-term effect of the approach on weight loss and metabolic changes was not studied.

Jensen et al [33] found that semaglutide is more effective than liraglutide in decreasing weight among participants who regained weight following BS. Nevertheless, the lack of randomization in their retrospective study made it susceptible to confounding bias. Beyond that, because their definition of *weight regain* after BS was not based on a consensual definition, they enrolled patients with any weight regain after BS, and data on the patients’ weight on liraglutide vs semaglutide were not differentiated. Such approaches increase the risk of selection bias because the weight loss effect of liraglutide and semaglutide could be affected by the amount of weight regain after surgery. Moreover, the patients had undergone different types and varying numbers of rounds of BS, with different time spans between BS and the initiation of GLP-1 RAs, all of which limits the generalizability of the study's findings.

Thakur et al [24] study showed favorable outcomes for liraglutide group after LSG in terms of weight reduction and metabolic disorders, it was limited by its small sample size and a short follow-up period of 6 months. Longer follow-up is needed to evaluate liraglutide's effect on weight reduction and metabolic disorders because the average time of plateau for liraglutide has been reported to occur at week 52 [49]. Moreover, the significant beneficial outcomes observed in relation to diabetes and prediabetes could be attributed to liraglutide as a treatment for diabetes. Last, their article did not mention the percentage of post-GLP-1 weight regain [24].

In Miras et al's study [26], liraglutide's effect on glucose and weight loss following metabolic surgery was found to be similar to that among patients with type 2 diabetes who did not have metabolic surgery. However, several limitations emerged in that study. The authors followed up with the patients for a short period (26 weeks), and the long-term benefits of GLP-1 RAs on glycemic control and weight loss were not tested because the plateau effect of those medications tends to start after 52 weeks [49]. GLP-1 RAs were initiated at least 12 months after metabolic surgery, which might have prompted the inclusion of patients who had lost a significant percentage of the benefits of metabolic surgery, meaning they had become somewhat similar to individuals who had not undergone the surgery. Added to that, only patients with persistent diabetes after bariatric surgery were enrolled and 1.8 mg liraglutide daily was used (the licensed maximal diabetes dose), although a higher dose of liraglutide is known to achieve a better weight reduction effect. That limitation may explain the nonsignificant difference between the study's intervention and placebo groups in terms of other metabolic outcomes, including hypertension and dyslipidemia. Last, the number of patients who had undergone VSG in the study was significantly less than that of patients who had undergone BS (ie, 11 and 42, respectively), which limits the generalizability of the findings to VSG.

The results of Wharton's retrospective study [23] showed that 3 mg liraglutide daily can achieve significant weight loss in post-BS patients with inadequate weight loss and/or weight regain. The limitations of the study included its retrospective design and lack of a control group. In addition, the researchers could not assess medication adherence or dose titration information, which might have resulted in variability in the weight loss reported. The study also included patients who had taken liraglutide for 4 months or less (47%, n = 55). Although current guidelines suggest the discontinuation of liraglutide if it does not achieve weight loss of at least 5% after 4 months, the inclusion of such patients might have meant the inclusion of nonresponders to liraglutide. Excluding those patients would have increased the percentage of patients who experienced clinically significant weight loss, namely from 41.9% to 59.4% [23].

Suliman et al [25] reported that 3 mg liraglutide can be a useful adjunctive treatment in patients who do not respond optimally to BS or regain weight thereafter. Nonetheless, their study had some limitations that may affect the reliability of their findings. First, all of the data included in their study (eg, medication adherence, side effects) were collected exclusively from medical records, which generally increases the risk of information bias. For another, the number of participants who had undergone BS was small (ie, 9%), and there was a significant gap between BS and the commencement of liraglutide—4 years—which may explain the nonsignificant difference in weight loss between the postsurgical and nonsurgical groups. Liraglutide's effect on other metabolic disorders, including hypertension, dyslipidemia, and atherosclerotic risks, was not studied. Beyond that, liraglutide's effect on weight loss was studied over 4 to 8 months only, and its long-term effect was not evaluated. The generalizability of that study's results is also limited by its inclusion of participants of Arab descent only, most of whom were female (77%).

In Rye et al's study [27], liraglutide decreased weight to a value less than the peak postsurgical weight in 50% of participants at 28 weeks. Early responders at 16 weeks were found to have better weight loss at 28 weeks. That difference may correlate with the type of surgery performed. Their study was limited, however, by being a retrospective study with a small sample of only 20 patients, a short follow-up period, and wide time variation between surgery and the liraglutide intervention (ie, 76.3 ± 72.9 months).

Liraglutide's effect on HbA1c reduction and weight loss was significant among patients with and without BS in Gorgojo-Martínez et al's study [50]. The average time between liraglutide's administration and BS was 5.2 years, which might explain the lack of difference between the BS and non-BS groups. The dropout rate was high in the non-BS group (34.9%), and the authors do not specify whether an intention-to-treat analysis was conducted. The maximum liraglutide dose used was 1.8 mg/d, which could explain the limited changes in the other metabolic outcomes (ie, lipid profile and blood pressure).

Last, Pajecki et al found significant weight loss reduction in patients who failed BS and received liraglutide. However, their study had a small sample size, and patients underwent different types of surgery, took liraglutide for different amounts of time, and were observed for periods that varied in length, all of which limit the generalizability of the study's findings.

### Limitations of Studies Examining EBT Plus Liraglutide

In Badurdeen et al's study [20], patients who received liraglutide following ESG had a significantly higher percentage of total body weight loss and body fat composition. However, the dose of liraglutide used in their study is not mentioned, and only a small sample of patients was included (ie, 30 participants received liraglutide). Moreover, the study was prone to recall and confounding biases given its retrospective data collection.

The results of Mosli et al's study [22] showed no advantages of administering liraglutide after the IGB insertion. Liraglutide was commenced 1 month after IGB insertion and stopped 1 month following IGB removal. Although the mean weight loss at the time of IGB removal was higher among patients in the IGB plus liraglutide group than in the IGB-only group, the authors attributed that outcome to the effect of other covariates, the details of which are not discussed in detail in their article. Even so, the authors concluded that there was a higher probability of treatment success in the IGB group after 6 months than in the IGB plus liraglutide group. The retrospective design of that study increased its risk of selection bias, recall bias, and confounding bias as well.

## Conclusion

The need for a safe, effective approach to managing obesity increases in parallel to the increasing prevalence of obesity and its complications. In our review, we compared the weight loss effect of adding liraglutide therapy to EBT and BS. A total of 11 studies was reviewed: 9 examining liraglutide plus BS and 2 examining liraglutide plus EBT. Combining liraglutide with EBT or BS achieved significant weight loss when compared with EBT or BS alone. Gastrointestinal symptoms were the most commonly reported side effects. Liraglutide's weight loss effect when combined with EBT was found to be comparable to the net weight loss (ie, nadir weight loss after BS-regained weight) following BS alone. However, metabolic changes were not assessed in studies examining liraglutide with EBT, and the inconsistency and heterogeneity between the included studies limited our ability to perform quantitative analysis. Overall, our review reveals a promising approach for managing obesity by combining GLP-1 RAs with EBT. The approach is expected to achieve shorter hospital stays, fewer side effects, and longer-term weight loss benefits than BS. However, additional prospective studies with higher-quality, more consistent outcome measures for weight loss and metabolic changes are needed to further evaluate the approach.

## Data Availability

Original data generated and analyzed in the study are included in this article or in the data repositories listed in the References.

## Abbreviations

BMI: body mass index
BS: bariatric surgery
EBT: endoscopic bariatric therapy
ESG: endoscopic sleeve gastroplasty
FDA: Food and Drug Administration
GB: gastric band
GLP-1 RA: glucagon-like peptide 1 receptor agonist
HbA1c: glycated hemoglobin
IGB: intragastric balloon
IQR: interquartile range
LSG: laparoscopic sleeve gastrectomy
RCT: randomized controlled trial
RYGB: Roux-en-Y gastric bypass
VSG: vertical sleeve gastrectomy
